# Data Mining–Based Model for Computer-Aided Diagnosis of Autism and Gelotophobia: Mixed Methods Deep Learning Approach

**DOI:** 10.2196/72115

**Published:** 2025-08-13

**Authors:** Mohamed Eldawansy, Hazem El Bakry, Samaa M Shohieb

**Affiliations:** 1 Information Systems Faculty of Computers and Information Mansoura University Mansoura Egypt

**Keywords:** social anxiety, emotion recognition, machine learning, facial expression analysis, neurodevelopmental disorders, GELOPH<15>, residual network with 50 layers, ResNet-50, artificial intelligence

## Abstract

**Background:**

Gelotophobia, the fear of being laughed at, is a social anxiety condition that affects approximately 6% of neurotypical individuals and up to 45% of those with autism spectrum disorder (ASD). This comorbidity can significantly impair the quality of life, particularly in adolescents with high-functioning ASD, where the prevalence reaches 41.98%. Accurate and automated detection tools could enhance early diagnosis and intervention.

**Objective:**

This study aimed to develop a deep learning–based diagnostic system that integrates facial emotion recognition with validated questionnaires to detect gelotophobia in individuals with or without ASD.

**Methods:**

The system was trained to identify ASD status using a balanced dataset of 2932 facial images (n=1466; 50% from individuals with ASD and n=1466; 50% from neurotypical individuals). The images were processed using the DeepFace library to extract facial features, which were then used as input for the deep learning classifier. After identifying ASD status, the same images were further analyzed using the pretrained DeepFace model to evaluate facial expressions for signs of gelotophobia. In cases where facial cues were ambiguous, the GELOPH<15> questionnaire, consisting of 15 items, was administered to confirm the diagnosis The system was fully implemented using the Python programming language. Deep learning models were developed using libraries such as PyTorch for training the multilayer perceptron classifier, while CUDA was used to accelerate computations on compatible graphics processing units. Additional libraries from the Python programming language, such as *scikit-learn, NumPy*, and *Pandas*, were used for preprocessing, model evaluation, and data manipulation. DeepFace was integrated using its Python application programming interface for facial recognition and emotion classification.

**Results:**

The dataset comprised 2932 facial images collected from platforms such as Kaggle and ASD-related websites, including 1466 (50%) images of children with ASD and 1466 (50%) images of neurotypical children. The dataset was split into 2653 (90.48%) training samples and 279 (9.51%) testing samples, with each image contributing 100,352 extracted features. We applied various machine learning models for ASD identification. The system achieved an overall prediction accuracy of 92% across both training and testing datasets, with the multilayer perceptron model demonstrating the highest testing accuracy. The system successfully classified gelotophobia in cases where facial expressions were clear. However, in cases of ambiguous facial cues, the DeepFace model alone was insufficient. Incorporating the GELOPH<15> questionnaire improved diagnostic reliability and consistency.

**Conclusions:**

This study demonstrates the effectiveness of combining deep learning techniques with validated diagnostic tools for detecting gelotophobia, particularly in individuals with ASD. The high accuracy achieved highlights the system’s potential for clinical and research applications, contributing to the improved understanding and management of gelotophobia among groups considered socially vulnerable. Future research could expand the system’s applications to broader psychological assessments.

## Introduction

### Background

Individuals with gelotophobia hold the belief that they are being laughed at. Over time, this belief may become internalized, resulting in heightened sensitivity to rejection, feelings of shame, and an increased tendency toward social avoidance. These observations highlight the complex relationship among laughter, social perception, and emotional regulation, particularly for those who struggle with interpreting subtle social cues. Laughter and humor are widely regarded as positive social tools that enhance interpersonal relationships and help individuals navigate life’s challenges, often evoking joy and connection. However, it is important to recognize that laughter can also carry negative connotations, particularly when used to mock or ridicule others [1]. For some individuals, distinguishing between different types of laughter is challenging. They may perceive all laughter as negatively directed at them, interpreting it as a form of mockery or threat, which often leads to social withdrawal and isolation [2]. While many people can manage feelings such as embarrassment, anger, or discomfort in response to ridicule, individuals with social competence challenges frequently struggle to regulate these emotions. This difficulty may foster a persistent fear of social interactions, rooted in the belief that they are being mocked for perceived social flaws [3,4].

### Autism Spectrum Disorder

Autism spectrum disorder (ASD) is a neurodevelopmental disorder that typically manifests in early childhood and persists throughout an individual’s life. The term *spectrum* reflects the wide variation in the severity of symptoms and abilities among individuals with ASD. Symptoms commonly include difficulties in social interactions, communication impairments, repetitive behaviors, and restricted interests. These challenges can range from mild to severe, influencing the individual’s ability to function in society [5]. ASD is diagnosed early, usually between 18 and 24 months, when signs such as delayed speech, limited social interactions, and unusual behavior patterns become apparent. In some cases, children may exhibit typical developmental patterns during the first year but later show regression, notably in social engagement and language skills [6]. According to a study by Harris [7], individuals with ASD often face difficulties in interpreting emotional expressions, which may hinder their ability to respond appropriately in social situations, leading to increased social withdrawal. Consequently, early diagnosis and intervention, including therapies such as applied behavior analysis, are crucial for improving outcomes. Recent advancements in data mining and machine learning are revolutionizing ASD diagnosis and management. Using deep learning models and facial expression recognition systems, the detection of subtle behavioral markers in individuals with ASD can be enhanced, facilitating earlier and more accurate diagnoses [8].

### Gelotophobia: Fear of Being Laughed at

Gelotophobia, the fear of being laughed at, is often linked to social anxiety disorders and can profoundly impact an individual’s psychological well-being. While laughter is generally perceived as a positive social signal, individuals with gelotophobia may interpret it as mockery or ridicule, even when no negative intent exists [9]. This heightened sensitivity to humor and social interactions is especially prevalent in individuals with ASD, who struggle to decode facial expressions and contextual cues in social environments [10]. Individuals with gelotophobia experience significant distress and may avoid social situations altogether to prevent perceived humiliation. This social withdrawal can exacerbate feelings of isolation and lead to further deterioration of mental health. The relationship between gelotophobia and autism is particularly significant because individuals with ASD often exhibit impaired ability to differentiate between various social cues, including laughter, making them more vulnerable to developing gelotophobia [11]. Research by Führ et al [12] suggests that approximately 45% of individuals with ASD are diagnosed with gelotophobia, highlighting the need for early detection and intervention. Unlike typical social anxieties, gelotophobia specifically affects the individual’s ability to engage socially due to constant fear of being laughed at or mocked. This fear can be debilitating, leading to severe social withdrawal and heightened anxiety.

### Link Between Gelotophobia and ASD

Gelotophobia is particularly prevalent in individuals with ASD due to their difficulties in understanding social cues and interpreting emotions. Studies show that individuals with ASD are more likely to misinterpret facial expressions, especially in humorous contexts, making them prone to developing gelotophobia [13]. For instance, they may interpret others’ laughter as mockery even when not intended harmfully. This misinterpretation can trigger intense embarrassment, shame, and anxiety. The heightened prevalence of gelotophobia among individuals with ASD stems not only from challenges interpreting social cues but also from increased sensitivity to negative social stimuli. Research by Husseiny et al [14] indicates that up to 41.98% of adolescents with high-functioning autism experience moderate gelotophobia. This statistic underscores the importance of recognizing and addressing this comorbidity to improve individuals’ quality of life.

Several studies have explored gelotophobia prevalence in individuals with ASD, indicating that it is a significant yet often overlooked aspect of autism. For example, research by Führ et al [12] found that individuals with ASD are more likely to develop gelotophobia due to limited ability to distinguish between benign and mocking laughter. These individuals, particularly with high-functioning autism, exhibited increased social anxiety, especially when perceiving themselves as ridicule targets. Ul Haque and Valles [15] emphasize the role of social context in gelotophobia development, highlighting heightened sensitivity to social interaction nuances. Negative childhood experiences, such as bullying or exclusion, may exacerbate this sensitivity, leading gelotophobia to develop as a defense mechanism.

### Research Gap and Study Objectives

While previous studies have examined the prevalence of gelotophobia in individuals with ASD and their challenges in interpreting social cues, objective and automated tools that integrate facial expression analysis with psychometric assessments remain lacking, limiting opportunities to improve early diagnosis and intervention. Current diagnostic methods often rely on subjective clinical observations, delaying identification and treatment. This study addresses the gap by proposing a novel deep learning–based model combining facial recognition (using DeepFace; Facebook AI Research) with the GELOPH<15> questionnaire, aiming to enhance accuracy and efficiency in detecting gelotophobia in children with ASD. This integrated approach offers a more objective and scalable solution to support clinical decision-making and improve patient outcomes.

Despite the known challenges of diagnosing gelotophobia and ASD via traditional clinical assessments and psychometric questionnaires, these methods rely heavily on subjective interpretation, often delaying timely diagnosis and intervention. Our study proposes an integrated approach combining DeepFace, a state-of-the-art facial recognition system that objectively analyzes subtle facial expressions, with the GELOPH<15> questionnaire, which captures social behaviors related to gelotophobia. This integration enables a comprehensive evaluation by leveraging behavioral self-reports and automated facial analysis.

Specifically, outputs from DeepFace, which detects nuanced facial cues indicative of gelotophobia and ASD traits, are used alongside questionnaire results to improve diagnostic accuracy. This method functions synergistically, enabling robust screening and early detection.

By applying a deep learning model with carefully selected hyperparameters, the study aimed to optimize prediction performance while ensuring generalizability across diverse populations. This artificial intelligence (AI)–powered diagnostic tool promises to reduce clinical burdens; provide objective assessment measures; and ultimately facilitate earlier, more effective interventions for children experiencing gelotophobia and ASD.

### The Role of AI and Deep Learning in Early Diagnosis

With advances in AI and machine learning, early ASD diagnosis is increasingly feasible through facial pattern analysis. Neural networks detect facial expressions linked to neurological conditions. Data mining systems are now being developed to aid in the diagnosis of both gelotophobia and ASD. Ganesan et al [16] found that 41.98% of adolescents with high-functioning autism experienced moderate gelotophobia, with female individuals showing higher rates, potentially due to increased bullying exposure during adolescence. This highlights the ASD-gelotophobia connection, suggesting that individuals with ASD may be more vulnerable to fear of being laughed at.

### Deep Learning Model for Detecting Gelotophobia and ASD

This paper introduces a deep learning model that analyzes facial features in children with ASD and typically developing children, aiming to achieve more accurate ASD identification. The model extracts facial characteristics and applies deep face analysis to predict gelotophobia. In addition, the GELOPH<15> questionnaire [17], consisting of 15 social behavior questions, has been developed as an interactive tool to detect gelotophobia.

### Literature Review

Brauer and Proyer [1] applied a deep convolutional neural network (CNN) to analyze facial expressions in images. The Facial Expression Recognition 2013 dataset, consisting of thousands of labeled facial expression images, was used for training. The CNN was structured to recognize subtle differences in facial expressions, which can be challenging for children with autism to interpret. The high accuracy rate (89.2%) indicates that CNNs can effectively support the development of tools designed to teach facial recognition skills to children with autism. This has significant implications for educational and therapeutic applications, providing a technological supplement to traditional methods. Further research could involve real-time applications of this model in interactive settings, such as games or virtual reality environments, to enhance engagement and learning outcomes for children with autism.

Canestrari et al [2] compared multiple machine learning models using the Visual Geometry Group (VGG)-16 architecture, a deep CNN pretrained on a large image dataset. By adapting VGG-16 for ASD prediction, they tested models such as support vector machine, CNN, and Haar cascade classifiers. Achieving 90% accuracy suggests that deep learning models, particularly VGG-16, can be instrumental in early diagnosis and intervention planning for ASD. The comparative approach also helps identify which models are most effective in clinical settings. Future work might focus on integrating these models into diagnostic tools that clinicians can use during routine checkups, potentially combining image data with other behavioral indicators for a more comprehensive assessment. Similarly, Arru et al [18] explored visual behavior analysis for ASD identification, highlighting the role of computer vision techniques in supporting early diagnosis.

On the other hand, Eslami et al [17] proposed AD-Diagnet, which integrated an autoencoder for unsupervised feature learning with a single-layer perceptron for classification. The use of functional magnetic resonance imaging data from the Autism Brain Imaging Data Exchange dataset allowed for the exploration of neural activity patterns associated with ASD. With an 82% accuracy, this approach highlights the potential of combining neural network models for extracting meaningful features from complex brain imaging data. It underscores the role of advanced machine learning techniques in improving the accuracy of neurodevelopmental disorder diagnoses. Expanding the framework to include additional layers or integrating it with other neural network architectures could further enhance accuracy. In addition, applying this model to other neuroimaging datasets could validate its generalizability and robustness.

The research by Leader et al [4] focused on eye-tracking data to assess visual attention in individuals with ASD. Unlike other studies that consider object semantics, this study specifically looked at the regions of the face that individuals with autism focus on during social interactions. The tendency of individuals with ASD to focus more on the mouth than the eyes provides crucial insights into the unique social processing characteristics of this population. This could inform the development of more targeted social skills training programs. Further studies could explore intervention methods aimed at encouraging eye contact in individuals with autism, as this may enhance social interaction and communication skills.

Moreover, Nunes et al [5] tracked eye movements in children during face recognition tasks, examining how different visual scanning patterns could be used to classify ASD. Machine learning models were developed to analyze these patterns. The findings, with an accuracy of 88.51%, suggest that eye movement patterns are a reliable marker for ASD. This could lead to the development of noninvasive, eye-tracking–based diagnostic tools that are easy to implement in various settings. Broader studies with larger, more diverse populations could enhance the generalizability of these findings. Moreover, integrating eye-tracking data with other behavioral and genetic data could improve the predictive power of ASD diagnostic tools.

Redfield et al [6] constructed a large-scale video dataset capturing social interactions in children to predict ASD. The analysis was conducted in 2 phases: detecting behaviors indicative of ASD and using these data for prediction through deep learning models. The use of real-world video data enhances the ecological validity of the findings, making the predictions more applicable to everyday settings. This study highlights the potential of video-based analysis for large-scale ASD screening. Future work should focus on enhancing deep learning models to accommodate diverse datasets, ensuring greater applicability across various cultural and demographic groups. In addition, developing real-time analysis tools that can be used in schools or clinics would be a significant advancement.

On the other hand, a study by Harris [7] reviewed various machine learning algorithms applied to diagnose ASD and learning disabilities using a dataset of infants’ videos. The study emphasized behaviors such as gaze, smile, and vocalization. The ability to use simple behavioral markers for early diagnosis underscores the potential for early intervention, which can significantly improve developmental outcomes for children with ASD. Future studies could enhance the model’s predictive accuracy by incorporating multimodal data, such as audio and physiological signals, alongside video analysis. Expanding the dataset to include a wider age range and more diverse populations would also be beneficial.

Alsaade and Alzahrani [8] proposed a transfer learning–based framework for facial recognition to improve autism detection. Transfer learning allowed the model to leverage preexisting knowledge from related tasks to enhance its performance. The framework’s 91% accuracy indicates that transfer learning can significantly improve the detection of autism, especially in environments with limited data. This makes it a practical solution for early detection in domestic settings. Implementing this framework in mobile apps or smart home devices could provide parents and caregivers with accessible tools for monitoring developmental milestones and identifying early signs of autism.

Titze [9] used eye-tracking technology in virtual reality environments to distinguish between children with autism and typically developing children, focusing on how visual attention is allocated during social tasks. With an accuracy of up to 86%, the findings suggest that eye-tracking can be a powerful tool for diagnosing ASD. Furthermore, the use of virtual reality environments offers a controlled setting for assessing social attention in a way that mirrors real-world interactions. Scaling up the sample size and including external validation datasets could strengthen the reliability of the findings. Exploring the use of virtual reality headsets to make eye-tracking more immersive and accessible is another promising direction.

The study by Ruch [10] explored the prevalence of gelotophobia among adolescents with high-functioning ASD. Using psychometric assessments, the research measured the fear of being laughed at and analyzed demographic factors. The findings of a 41.98% prevalence rate highlight the importance of addressing emotional and psychological aspects in the treatment of ASD. This study also points to gender differences in the experience of gelotophobia, which could inform more tailored interventions. Further research could examine the underlying causes of gelotophobia in those with ASD and explore interventions that reduce social anxiety. Longitudinal studies tracking these adolescents into adulthood could provide insights into how gelotophobia evolves over time and its long-term impacts (Table 1).

**Table 1:** Summary of related work on autism spectrum disorder (ASD) detection using machine learning (ML) and other methods.

Study	Focus area	Dataset	Algorithm	Accuracy (%)		Age range	GELOPH<15> test
Ul Haque and Valles [15], 2018	Using smartphones or tablets to help children with autism recognize facial expressions	Kaggle Facial Expression Recognition 2013 dataset (web based)	DCNN^a^	89.2	Not specified		Not used
Ganesan [16], 2021	Classification of ASD in children using ML for facial analysis	Kaggle repository (web based)	VGG-16^b^	90	Not specified		Not used
Eslami et al [17], 2019	Developing ASD-DiagNet to classify ASD using fMRI^c^ data	ABIDE^d^ dataset (web based)	ASD-DiagNet	82	Not specified		Not used
Liu et al [19], 2016	Classifying ASD in children based on atypical face scanning patterns (eye movement data)	29 children diagnosed with ASD (local dataset)	SVM^e^	88.51	Not specified		Not used
Wu et al [20], 2021	Early ASD diagnosis using ML to analyze infant behaviors from video data	Interactive play session video dataset (web based)	Baseline ML classifier	82	Infants (0-3 y)		Not used
Akter et al [21] 2021 and Alcañiz et al [22], 2022	Transfer learning–based facial recognition framework for early ASD detection	Kaggle data repository (web based)	MobileNet-V1	92.1	Children (4-12 y)		Not used
Kats [23], 2022	Eye-tracking in virtual reality to distinguish ASD from typically developing children	Kaggle data repository (web based)	SVM	86	Children (3-12 y)		Not used
Husseiny et al [14], 2024	Examining gelotophobia prevalence and severity in adolescents with high-functioning ASD	Real-world data from Cairo Governorate, Egypt	EFA^f^ and CFA^g^	76.88% variance explained, with excellent fit indices (*χ*^2^=59.4 and RMSEA^h^=0.000)	Adolescents (13-18 y)		Used (after study)

^a^DCNN: deep convolutional neural network.

^b^VGG-16: Visual Geometry Group 16.

^c^fMRI: functional magnetic resonance imaging.

^d^ABIDE: Autism Brain Imaging Data Exchange.

^e^SVM: support vector machine.

^f^EFA: exploratory factor analysis.

^g^CFA: confirmatory factor analysis.

^h^RMSEA: root mean square error of approximation.

### Study Contribution

This study developed a dual diagnostic framework aimed at the early detection of both ASD and gelotophobia. The proposed system achieved a high classification accuracy of 92% for ASD using a deep learning–based facial emotion recognition model (multilayer perceptron [MLP]). To improve the reliability of gelotophobia detection, especially in ambiguous cases, the framework integrated facial expression analysis using the DeepFace model with the GELOPH<15> psychometric questionnaire. This hybrid approach effectively combines objective facial analysis with subjective questionnaire evaluation, allowing simultaneous assessment of multiple psychological conditions. The study used a publicly available, balanced dataset of 2932 images, ensuring reproducibility and practical applicability. Furthermore, it offers a scalable and comprehensive tool to support early diagnosis and intervention planning for populations considered socially vulnerable. Finally, the framework lays a foundation for future research in expanding multimodal behavioral markers, such as voice patterns and body language.

## Methods

### Proposed Framework and Methods

**Figure 1:**
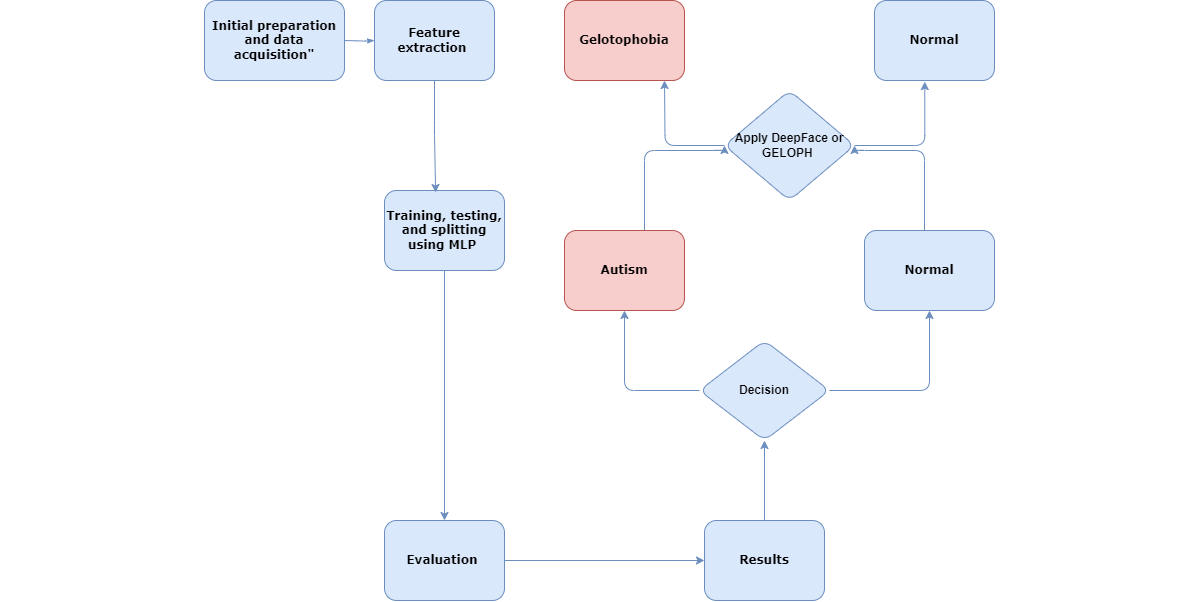
Workflow of the proposed gelotophobia prediction framework integrating DeepFace, GELOPH<15> questionnaire, and multilayer perceptron (MLP).

This study proposed an integrated framework to predict gelotophobia in both individuals with and without autism using facial analysis and questionnaire data. The methodology combined deep learning DeepFace and residual network with 50 layers (ResNet-50), psychological assessment (GELOPH<15>), and a classification model (MLP). Figure 1 illustrates the overall workflow of the framework.

### Dataset

This study examined facial photographs of children with ASD and typical children taken from the publicly available web-based Kaggle platform [11]. The dataset comprised 2932 facial photographs: half (n=1466, 50%) of the photographs showed children without ASD, while the other half (n=1466, 50%) depicted children with autism. This information was gathered from online resources, including ASD-related web pages and Facebook (Meta Platforms, Inc) pages. The training data contained 2653 (90.48%) records, each with 100,352 features (float64), and the test data had 279 (9.51%) records. The shape of the target vectors suggested a binary classification problem, as there were 2 distinct class types in both training and test sets.

### Initial Preparation

The photographs were cleaned and cropped as part of the data preparation. The data had to be preprocessed before they could be used to train the deep learning model, as Piosenka gathered them from web-based sites. The face in the original image was automatically cropped by the dataset author. The dataset of 2932 photographs was divided into 279 (9.51%) photographs for testing and 2653 (90.48%) images for training. The normalization approach was used to scale, with all picture parameters being rescaled from (0, 255) to (0, 1) in the dataset.

### Feature Extraction

#### Overview

A CNN design, ResNet-50, is an integral component of the residual network (ResNet) family. Because of its well-known deep architecture, ResNet succeeds in challenges involving picture recognition and classification. In particular, ResNet-50 is a 50-layer version of the ResNet architecture that is comparatively powerful and deep [24].

#### Key Features of the ResNet-50 Architecture

**Figure 2:**
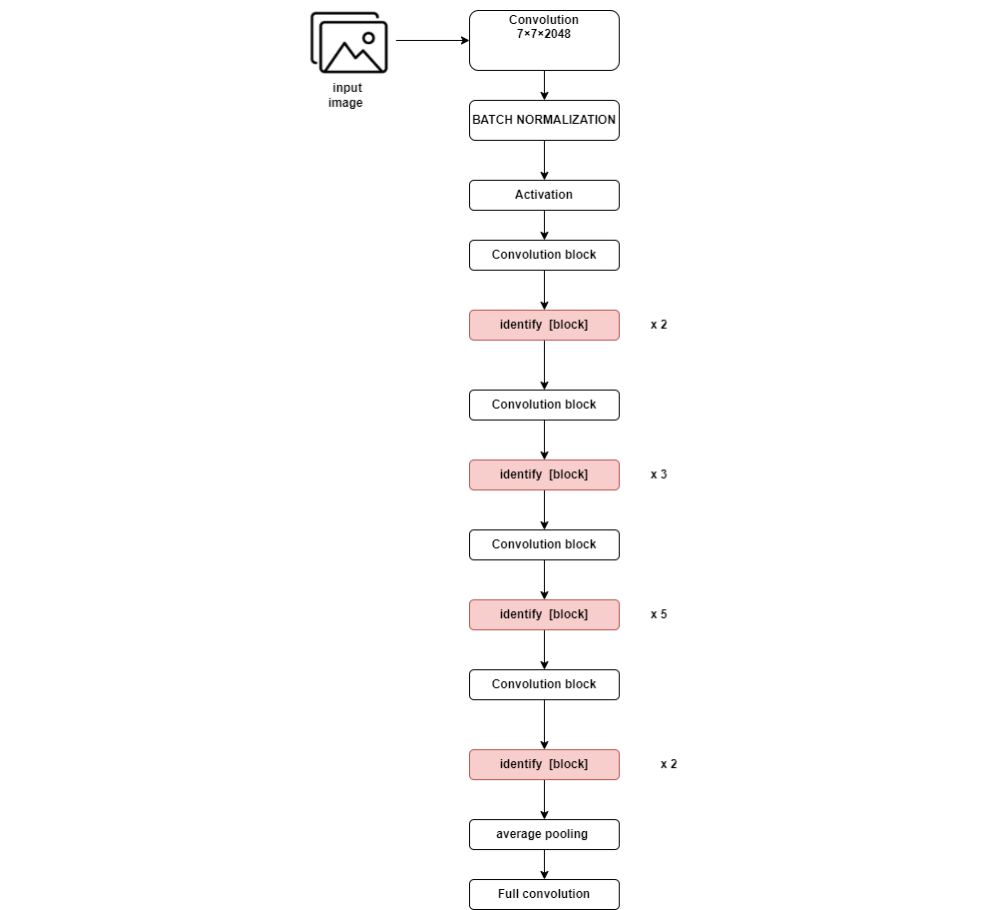
Residual network with a 50-layer architecture used for feature extraction.

ResNet-50 is a deep learning model consisting of 50 layers, which include 1 convolutional layer, 16 residual blocks (each containing multiple convolutional layers), a global average pooling layer, and a fully connected (dense) layer. A major innovation of ResNet-50 is its use of residual blocks with identity shortcut connections that bypass certain layers, addressing the vanishing gradient issue commonly encountered during deep network training. Furthermore, the model uses a bottleneck design within the residual blocks to optimize computation, incorporating 3 convolutional layers: a 1×1 convolution to reduce dimensions, a 3×3 convolution for the core transformation, and a second 1×1 convolution to restore the original dimensions. With approximately 23 million parameters, ResNet-50 is more computationally efficient than deeper variants such as ResNet-101 and ResNet-152. This architecture has demonstrated high performance in tasks such as object detection, image segmentation, and image classification. ResNet-50 notably triumphed in the 2015 ImageNet competition, achieving significantly lower training errors than previous models. This model, presented in Figure 2, processed images and extracted features from them using the network. The extracted features, along with their corresponding labels, were stored in CSV files for later use with traditional machine learning algorithms. In addition, the label encoder was saved for decoding the labels in the future.

### MLP Model

MLP was a kind of artificial neural network that was exploited in deep learning and machine learning. Because it was a feedforward neural network, there were no loops or feedback connections in the information flow, which proceeded from the input layer through hidden layers to the output layer.

#### Input Layer

The input features of the data were represented by the neurons (nodes) in this layer. The input values were given to each neuron, which represented a feature.

#### Hidden Layers

There might be 1 or more hidden layers in between the input and output layers. Every buried layer was made up of many neurons. To extract complicated patterns and representations from the input data, these neurons computed the data using activation functions and weights.

#### Weights

Weights were the parameters that the network learned during training. They verified how strongly neurons in various levels were connected to one another. The network learned to make predictions by varying these weights.

#### Activation Functions

Every neuron in the buried layers usually adds a weighted sum of its inputs to an activation function. The sigmoid rectified linear unit and hyperbolic tangent functions were examples of common activation functions. These functions provided the model with nonlinearity, which enabled it to discover complex links in the data.

#### Output Layer

Using the data acquired in the hidden layers, the output layer, which was the last layer, generated the model’s predictions. The kind of problems being solved determines how many neurons were in this layer. For multiclass classification, there were numerous neurons, 1 for each class; however, for binary classification, there was 1 neuron that outputs a probability.

### MLP Architecture and Configuration

**Figure 3:**
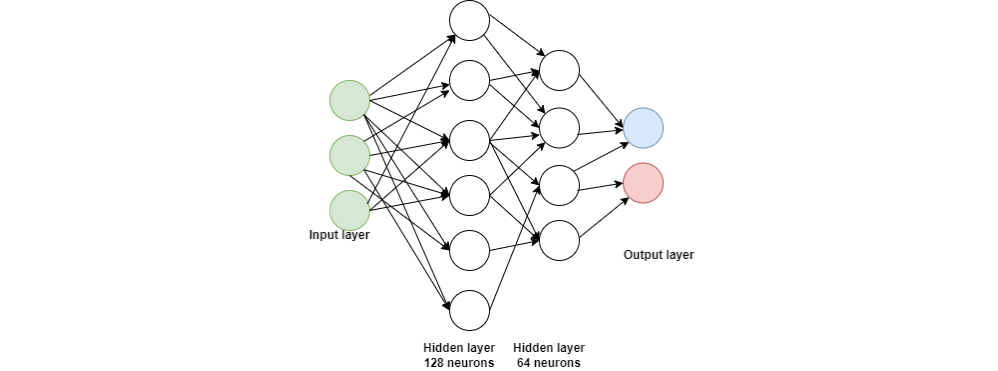
Architecture of the multilayer perceptron model used for autism spectrum disorder prediction.

The MLP model used in this study consisted of 2 hidden layers, each serving a distinct functional role in the feature learning process. The first hidden layer was composed of 128 neurons and was designed to capture high-dimensional and complex patterns from the input data. This larger capacity allowed the network to model intricate relationships that were often not linearly separable. The second hidden layer contained 64 neurons and was intended to refine the representations learned by the first layer. By reducing the dimensionality of the intermediate feature space, it retained only the most salient and relevant patterns necessary for effective classification. This hierarchical structure emulated human perceptual processes, where general features were recognized first, followed by more specific details. This model had 2 hidden layers, as shown in Figure 3. The MLP model was trained using the MLP classifier module from the scikit-learn library with the parameters provided in Table 2.

**Table 2:** Multilayer perceptron parameters.

Parameter	Description
Hidden layer sizes=(128, 64)	Two hidden layers with 128 and 64 neurons
Activation=“relu”	ReLU^a^ activation function for nonlinearity
Solver=“adam”	Adam^b^ optimizer for efficient training
Alpha=0.0001	L2 regularization coefficient to prevent overfitting
Batch size=64	Number of samples per gradient update
Learning rate=“constant”	Static learning rate throughout training
Initial learning rate=0.001	Initial learning rate for weight updates
Maximum iterations=1000	Maximum number of iterations for convergence
Momentum=0.9	Momentum for SGD^c^ (not used with Adam)
Nesterov momentum=true	Enables Nesterov momentum (if SGD is selected)

^a^ReLU: rectified linear unit.

^b^Adam: adaptive moment estimation.

^c^SGD: stochastic gradient descent.

The hidden layer sizes (128 and 64), learning rate (0.001), and batch size (n=64) were selected based on preliminary experiments that aimed at optimizing classification accuracy while avoiding overfitting. These values were also consistent with commonly adopted configurations in similar deep learning classification tasks.

Following training, the MLP model was used to classify the input images into autistic or nonautistic categories. The output of this classification was then passed to subsequent modules for gelotophobia analysis via facial expression recognition and questionnaire validation.

### Confusion Matrix

**Figure 4:**
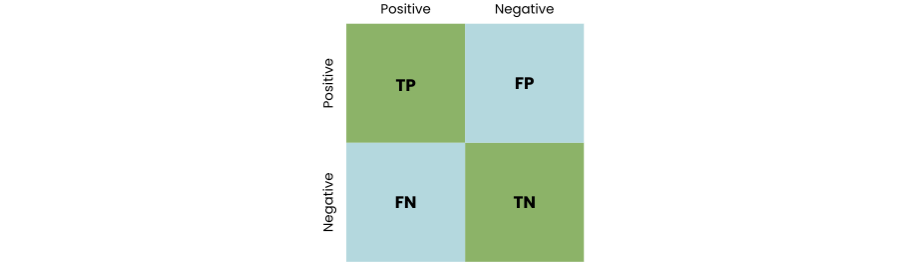
Confusion matrix. FN: false negative (incorrectly classified as negative when they are actually positive); FP: false positive (incorrectly classified as positive when they are actually negative); TN: true negative (correctly classified negative instances); TP: true positive (correctly classified positive instances).

Offering a tabular depiction of true and false values in test results, a confusion matrix is a useful tool for assessing categorization performance. The confusion matrix of the MLP model is provided in Figure 4.

Accuracy, defined as the percentage of correct classifications, was calculated using the following formula:

Accuracy = (TP + TN) / (FP + FN + TP + TN) × 100% **(1)**

Specificity, which measured the model’s ability to correctly identify children without autism, was computed as follows:

Specificity = TN / (TN + FN) × 100% **(2)**

Sensitivity, which assessed the model’s capability to accurately identify children with autism, was determined as follows:

Sensitivity = TP / (TP + FP) × 100% **(3)**

In equations 1 to 3, TP represents true positive, FP represents false positive, TN represents true negative, and FN represents false negative. Specificity focused on correctly identifying normal children, while sensitivity focused on correctly identifying children with autism.

### DeepFace

**Figure 5:**
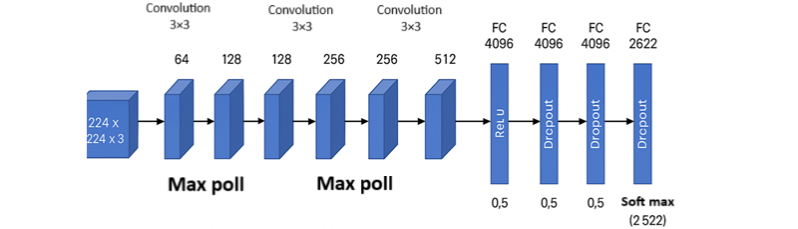
Architecture of the Visual Geometry Group (VGG) Face model used in DeepFace. FC: fully connected; ReLU: rectified linear unit.

DeepFace is a lightweight and versatile deep learning framework designed for face recognition and facial attribute analysis. It abstracts the complexity of multiple state-of-the-art face recognition models, providing a unified interface for tasks such as face verification, identification, and emotion analysis. DeepFace supports several backbone models, including VGG-Face, Facenet, ArcFace, Dlib, and more, allowing flexible deployment depending on application needs. Among these, VGG-Face served as the default model, recognized for its robust performance in face recognition tasks. The VGG-Face architecture, as depicted in Figure 5, was based on the VGG-16 network and consisted of a deep stack of convolutional layers followed by fully connected layers, culminating in a softmax classifier trained on a large-scale dataset of celebrity faces.

### Integration of DeepFace and GELOPH<15> Outputs

In this study, a sequential approach was used to integrate the outputs of the MLP, DeepFace, and GELOPH<15> models for diagnosing ASD and predicting gelotophobia. First, facial images were fed into the MLP model to classify whether the individual had ASD. Then, the same images were analyzed by DeepFace to detect facial expressions linked to gelotophobia. If DeepFace identified emotions associated with gelotophobia, the prediction was further validated using the GELOPH<15> psychometric questionnaire, which assessed self-reported fear of being laughed at. This hybrid approach combined objective facial expression analysis with subjective questionnaire responses to improve the accuracy and reliability of gelotophobia detection, especially in ambiguous cases.

### Ethical Considerations

This study was approved by the Faculty of Computers and Information Research Ethics Committee (approval code 2024 01 004). All procedures were conducted in accordance with the Declaration of Helsinki and followed ethical standards regarding data privacy and the responsible use of AI in health-related research. Facial image data used for autism detection were obtained from a publicly available, anonymized dataset on Kaggle, ensuring no personally identifiable information was used. The GELOPH<15> questionnaire was not directly administered to participants in this study; it was used solely as a reference from previously validated research. No direct contact with human participants occurred, and all data were either publicly available or ethically approved under the research protocol.

## Results

### Overview

The experimental results were obtained using a PC equipped with an 11th-generation Intel Core i7-11800H control processing unit running at 2.30 GHz, 16 GB of RAM, and a 64-bit Windows 10 operating system. The proposed system and experiments were implemented using Python 3.9.7 (Python Software Foundation), along with the following libraries: scikit-learn 0.24.2, TensorFlow 2.9.2, and DeepFace 0.0.93. The results of our research to identify ASD and gelotophobia are presented in this section. In this analysis, we performed 10-fold cross-validation on the training data using an MLP classifier to evaluate its performance on predicting the target variable. The cross-validation process allowed us to assess the model’s generalizability by dividing the data into 10 distinct subsets or folds, ensuring that each subset served as a validation set while the remaining data were used for training. For each fold, several evaluation metrics, including accuracy, precision, recall, and *F*_1_-score, were calculated. The results for each fold are provided in Table 3, along with the average and SD across all folds.

**Figure 6:**
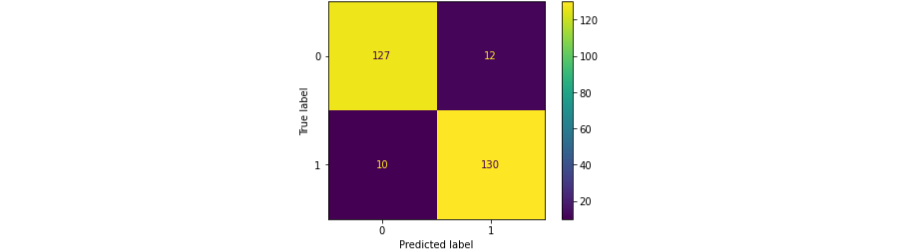
Confusion matrix.

The best model was then used for testing; the model’s performance was assessed on the test data using classification metrics and a confusion matrix, which were used for further evaluation. The system achieved 92% accuracy, 92% precision, 93% recall, and 92% *F*_1_-score. The confusion matrix is provided in Figure 6.

**Table 3:** Performance metrics of the multilayer perceptron classifier using 10-fold cross-validation for the detection of autism spectrum disorder and gelotophobia.

Fold	Accuracy	Precision	Recall	*F*_1_-score
1	0.800752	0.800905	0.800752	0.800727
2	0.815789	0.81595	0.815789	0.815766
3	0.793233	0.794048	0.793233	0.79309
4	0.796226	0.796226	0.796226	0.796226
5	0.781132	0.784392	0.781132	0.780563
6	0.803774	0.804073	0.803774	0.80374
7	0.773585	0.774186	0.773585	0.773488
8	0.822642	0.82358	0.822642	0.822535
9	0.818868	0.81893	0.818868	0.818852
10	0.830189	0.830373	0.830189	0.830174
All folds, average (SD)	0.803619 (0.018339)	0.804266 (0.017908)	0.803619 (0.018339)	0.803516 (0.018427)

**Figure 7:**
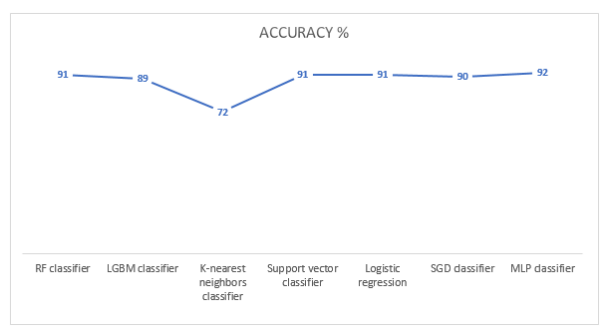
Comparison of classification models for autism spectrum disorder identification based on facial features. The table summarizes each model’s testing accuracy. LGBM: light gradient boosting machine; MLP: multilayer perceptron; RF: random forest; SGD: stochastic gradient descent.

For identifying ASDs, we compared the proposed system with various machine learning models to validate its performance, including a random forest classifier, light gradient boosting machine classifier, K-nearest neighbors classifier, support vector classifier, logistic regression, stochastic gradient descent classifier, and MLP classifier, as shown in Figure 7. Each model was trained and tested to detect facial characteristics distinguishing children with autism from those without autism. Notably, the MLP model outperformed all other models, achieving the highest testing accuracy of 92%. In contrast, the K-nearest neighbors classifier delivered the lowest accuracy at 72%. The dataset was generated from various internet sources, leading to natural variability in age and image quality. Nevertheless, the MLP model demonstrated exceptional accuracy and robustness, as provided in Table 4.

**Table 4:** Pretrained deep learning models.

Algorithm	Accuracy (%)	Precision (%)	Recall (%)	*F*_1_-score (%)
RF^a^ classifier	91	90	91	91
LGBM^b^ classifier	89	88	91	89
K-nearest neighbors classifier	72	64	98	77
Support vector classifier	91	89	94	91
Logistic regression	91	89	94	91
SGD^c^ classifier	90	92	88	90
MLP^d^ classifier	92	91	92	92

^a^RF: random forest.

^b^LGBM: light gradient boosting machine.

^c^SGD: stochastic gradient descent.

^d^MLP: multilayer perceptron.

### Evaluation Outcomes

A trained deep learning model called DeepFace was created by Facebook’s AI research and was built on different deep neural network architectures for face analysis tasks, including emotion detection. CNNs were commonly used by DeepFace and related models to extract features from facial photographs [13]. DeepFace is a lightweight face recognition and facial attribute analysis framework. It wraps several models, such as *VGG-Face.*

The model was based on the *VGG-16* CNN architecture (Table 5). Key features are as follows: the input was RGB (red, green, and blue) images of size 224×224; there were 13 convolutional layers, 5 max pooling layers, and 3 fully connected layers at the end; activation included rectified linear unit for all layers; and the final fully connected layer was softmax with 2622 classes (corresponding to 2622 people in the training set).

**Table 5:** DeepFace model training parameters.

Parameter	Value or description
Learning rate	Started at 0.001 (learning rate), decreased during training
Optimizer	SGD^a^
Momentum	0.9 (momentum)
Weight decay	5e-4 (0.0005; L2 regularization [ridge regression])
Batch size, n	256
Dropout	0.5 (dropout; in fully connected layers)
Training time	Trained on 2.6 M images for days using 4 GPUs^b^
Loss function	Softmax cross entropy

^a^SGD: stochastic gradient descent.

^b^GPU: graphical processing unit.

The deep learning model typically used ≥1 fully linked layers, often referred to as dense layers, to conduct classification after feature extraction. These layers took the characteristics that had been collected and used machine learning to associate them with particular emotions. Similar emotion identification challenges may be implemented with open-source deep learning frameworks such as PyTorch or TensorFlow. These frameworks provided the ability to design the architecture and equations for unique deep learning models, which may be used to construct and train models for a variety of tasks, including emotion detection.

Facial expressions indicating gelotophobia (the fear of being laughed at) included tense brows, forced smiles, avoiding eye contact, blushing, and nervous laughter, all of which reflected discomfort or the fear of being ridiculed. If a person’s face exhibited these signs, we can predict using an algorithm whether they had gelotophobia or not.

### GELOPH<15>

The GELOPH<15> test has proven to be a reliable tool for measuring gelotophobia among children and adolescents. In a study conducted with a sample of children and adolescents aged 11 to 16 years, the Danish version of the GELOPH<15> questionnaire demonstrated good psychometric properties, including high internal consistency and a 1D factor structure that closely aligned with the adult version. Despite the need for younger participants to receive assistance from teachers in filling out the items, the test was found to be reliable and easily understood by most participants. The study highlighted that the GELOPH<15> questionnaire can serve as a solid foundation for future research on gelotophobia, with its capacity to reliably measure the fear of being laughed at across various age groups. Furthermore, the GELOPH<15> questionnaire’s consistency with the adult version indicated its applicability across different populations, offering a robust tool for studying gelotophobia in both children and adults [14].

The GELOPH<15> consists of 15 items rated on a 4-point Likert scale (1 = strongly disagree to 4 = strongly agree). A mean score of 2.5 or higher typically indicates the presence of gelotophobia. If participants strongly agree with at least 4 statements, this may also reflect a strong tendency toward gelotophobia (Multimedia Appendix 1 [25]).

## Discussion

### Principal Findings

This study aimed to explore the potential of facial emotion recognition in detecting ASD and gelotophobia using deep learning approaches. Our system achieved a high accuracy of 92%, surpassing many existing models in the field. Integration the DeepFace model with an MLP classifier allows for robust feature extraction from facial expressions, contributing to a reliable ASD diagnosis. In addition, we included the GELOPH<15> questionnaire to assess gelotophobia, a comorbid condition often observed in individuals with ASD. This dual-focus approach of detecting ASD while simultaneously considering the social anxieties related to being laughed at offers a more comprehensive tool than previous models that typically focus on ASD alone.

The proposed system serves as a powerful support tool for clinicians in diagnosing ASD and gelotophobia, enabling faster and more accurate assessment compared to traditional methods. With each use, AI continuously learns from new data, improving its performance over time and enhancing the reliability of clinical decisions. This makes the system easily integrable into existing clinical care pathways, reducing the burden on medical staff and improving patient outcomes through earlier and more comprehensive diagnosis.

### Model Interpretability and Clinical Relevance

Although deep learning models, such as DeepFace and MLP, are often considered “black boxes,” we have taken steps to enhance their interpretability for clinical use. Key facial expression features related to emotions such as fear, sadness, and social anxiety were identified as the most informative indicators for classifying ASD and gelotophobia. To assist clinicians, our system can be extended to include visualization tools, such as heat maps or feature importance scores (eg, using Shapley additive explanation or local interpretable model-agnostic explanation techniques), which highlight the facial regions or features most influential in the model’s decisions. This transparency supports clinicians in understanding and trusting the system’s output, facilitating its integration into diagnostic workflows.

### Comparison to Previous Work

Table 6 compares the performance of our model with that of several notable studies in the field, focusing on accuracy, methodology, and the disorders targeted. As shown in Table 6, our system outperforms many previous approaches, especially in accuracy, while also addressing gelotophobia, which is often overlooked in ASD detection (Table 7).

**Table 6:** Comparison to previous work.

Authors	Model or approach	Accuracy (%)	Disorder targeted	Notes
This study	DeepFace and MLP^a^	92	Autism or gelotophobia	High accuracy, combining facial features and deep learning
Ul Haque and Valles [15], 2018	DCNN^b^	82	Autism	Lower accuracy; focused on facial expression detection
Ganesan et al [16], 2021	ML^c^	84.5	Autism	Focused on facial features; less robust generalization
Eslami et al [17], 2019	Hybrid learning (fMRI^d^)	90	Autism	High, but <92%; relies on fMRI, which limits practicality
Liu et al [19], 2016	ML on face processing	81.7	Autism	Insufficient accuracy for clinical use
Wu et al [20], 2021	ML from videos	89.2	Autism	Video-based study; strong results, but <92%
Canestrari et al [2], 2021	Survey or statistical analysis	N/A^e^	Gelotophobia (with ASD^f^)	Explores gelotophobia in those who experience cyberbullying with parental attachment correlations
Leader et al [4], 2018	Statistical analysis (psychometrics)	N/A	Gelotophobia in individuals with ASD	Psychological assessment of gelotophobia in individuals with high-functioning ASD
Husseiny et al [14], 2024	Questionnaire based	N/A	Gelotophobia in teenagers with ASD	Descriptive statistics; not a classifier

^a^MLP: multilayer perceptron.

^b^DCNN: deep convolution neural network.

^c^ML: machine learning.

^d^fMRI: functional magnetic resonance imaging.

^e^N/A: not applicable.

^f^ASD: autism spectrum disorder.

**Table 7:** Comparison of the results of our system and existing models.

Study	Deficiency
Ul Haque and Valles [15], 2018	Focused only on autism detection using the Facial Expression Recognition 2013 dataset, which lacks diversity in expressions, age, ethnicity, and gender
Ganesan et al [16], 2021	Limited to diagnosing autism, with challenges such as low SVM^a^ accuracy (approximately 65%) and dataset diversity affecting model generalizability
Eslami et al [17], 2019	Focused solely on autism detection using fMRI^b^ data, with accuracy (82%) insufficient for clinical application, and issues with generalization
Nunes et al [5], 2019	Preliminary research, not yet integrated into clinical practice; lacks robust multimodal fusion development
Wu et al [20], 2021	Low accuracy in detecting behavioral cues such as vocalization (53%) and object focus (67%), limiting ASD^c^ diagnosis reliability
Alsaade and Alzahrani [8], 2022	Transfer learning risks overfitting, especially on small or homogeneous datasets, affecting generalization
Ruch [10], 2009	Study limited by small sample size, lack of diversity, and reliance on self-reports, which may bias gelotophobia assessment in individuals with high-functioning ASD

^a^SVM: support vector machine.

^b^fMRI: functional magnetic resonance imaging.

^c^ASD: autism spectrum disorder.

### Limitations

Despite promising accuracy, our study has several limitations. First, the dataset—sourced from publicly available 2D facial images—may not fully represent the diversity of facial expressions in children with ASD or gelotophobia, as demographic variables, such as age, gender, and cultural background, were not explicitly controlled. Second, while 10-fold cross-validation mitigates overfitting, the lack of an external validation cohort limits confidence in generalizability. Third, subtle emotional states (eg, fear and disgust) remain challenging for the current model to distinguish reliably, as these expressions often involve minimal facial muscle movements. Fourth, our approach relies solely on static images; real-world interactions are dynamic and multimodal (including voice, gesture, and context), which our system does not capture. Finally, although we propose advanced architectures (ResNet and EfficientNet) and multimodal inputs (eye-tracking and speech) as future directions, these were not implemented in this study.

### Future Work

To address the current limitations, future efforts will focus on expanding and diversifying the dataset by collecting and annotating a larger, demographically varied sample of children with and without ASD and gelotophobia, followed by validating the model on independent cohorts from multiple institutions to ensure robustness. We also plan to experiment with more advanced model architectures such as deeper CNNs, including ResNet-50 and EfficientNet-B4, and lightweight models, such as MobileNet-V3, aiming to improve the extraction of subtle facial expressions. Furthermore, integrating multimodal data, including eye-tracking, speech prosody, and body language cues, will allow capturing dynamic emotional signals and reduce reliance on static images. The development of a real-time application prototype and conducting pilot studies in clinical settings will evaluate the system’s usability, acceptability, and diagnostic utility. In addition, longitudinal studies are planned to investigate whether early emotion recognition markers can predict later social outcomes or responses to interventions. Finally, we will incorporate explainable AI techniques to interpret model decisions and implement fairness checks to detect and mitigate demographic biases.

### Conclusions

This study demonstrated the promising potential of using facial expression recognition combined with deep learning techniques for the detection of ASD and gelotophobia. By leveraging the power of the DeepFace model to extract facial features, we achieved an accuracy of 92% using an MLP classifier. These findings highlight the significant role of facial expression analysis in identifying subtle emotional processing deficits in individuals with ASD and gelotophobia, both of which are often characterized by challenges in interpreting social cues.

The results emphasize that deep learning models, particularly those capable of processing facial expressions, can serve as valuable tools in autism diagnosis and the assessment of related comorbid conditions. This approach has the potential to aid clinicians by providing a noninvasive, reliable method for early detection and intervention.

However, further research is needed to refine the model, particularly in increasing its generalizability across diverse populations and enhancing its ability to detect a wider range of emotional expressions. In addition, exploring other advanced deep learning architectures, such as ResNet or EfficientNet, could further improve accuracy. The integration of multimodal data, including eye-tracking and voice analysis, might also provide a more comprehensive assessment of emotional processing in ASD and gelotophobia.

In summary, this study lays the groundwork for future advancements in the field of AI-based diagnostic tools for ASD and related conditions, paving the way for more efficient and accurate clinical practices.

## Appendix

Multimedia Appendix 1 Fear of being laughed at (gelotophobia) questionnaire.

## Abbreviations

AI: artificial intelligence
ASD: autism spectrum disorder
CNN: convolutional neural network
FP: false positive
FN: false negative
MLP: multilayer perceptron
ResNet: residual network
ResNet-50: residual network with 50 layers
TN: true negative
TP: true positive
VGG: Visual Geometry Group

## Data Availability

The datasets generated or analyzed during this study are available in the Kaggle repository [26].
