# Expression of Concern: Data Mining–Based Model for Computer-Aided Diagnosis of Autism and Gelotophobia: Mixed Methods Deep Learning Approach

**DOI:** 10.2196/91833

**Published:** 2026-01-23

Following the publication of this article [1] concerns were raised regarding the validity of the dataset used. The JMIR Publications Editorial Office is investigating this matter and in the interim issues this expression of concern.
