# Supplementary material for: Data Mining–Based Model for Computer-Aided Diagnosis of Autism and Gelotophobia: Mixed Methods Deep Learning Approach
Source: JMIR Form Res. 2025 Aug 13;9:e72115. doi: 10.2196/72115 (PMC12391841; doi:10.2196/72115)
Supplement: Multimedia Appendix 1 [file formative_v9i1e72115_app1.docx]

**Fear of Being Laughed At (Gelotophobia) – Questionnaire**

Please respond to each of the following 15 statements by indicating **“Yes”** if you agree or **“No”** if you do not agree. These items are used to assess the degree to which individuals fear being laughed at or ridiculed by others.

1. When people laugh in my presence, I become suspicious and think it might be about me.
2. I avoid showing myself in public because I fear others will notice my insecurity and make fun of me.
3. When strangers laugh near me, I often assume they are laughing at me.
4. I find it difficult to make eye contact because I fear being judged in a negative way.
5. When others make joking remarks about me, I feel frozen or paralyzed.
6. I try hard to control myself and my behavior so I don’t attract negative attention or look ridiculous.
7. I believe that I unintentionally make a funny or strange impression on others.
8. Even though I often feel lonely, I avoid social situations to protect myself from being mocked.
9. If I embarrass myself in a certain place, I tend to avoid going back there.
10. If I weren’t afraid of making a fool of myself, I would speak more often in public.
11. If someone has teased me in the past, I can’t interact freely with them anymore.
12. It takes me a very long time to recover emotionally after being laughed at.
13. I feel uncomfortable while dancing because I believe others see me as ridiculous.
14. I feel that unless I am very careful, I will attract negative attention and seem odd to others.
15. When I embarrass myself in front of others, I freeze up and can’t behave normally anymore.

**Reference**

Ruch, W., & Proyer, R. T. (2008). **The fear of being laughed at: Individual and group differences in gelotophobia**. *Humor: International Journal of Humor Research, 21*(1), 47–67. https://doi.org/10.1515/HUMOR.2008.002
